# Acute Amiodarone Pulmonary Toxicity Following Lung Resection

**Published:** 2014-09

**Authors:** Opeyemi Fadahunsi, Ronald Krol

**Affiliations:** 1 Department of Medicine, Reading Health System, West Reading, PA, USA;; 2 Division of Pulmonary and Critical Care, Reading Health System, West Reading, PA, USA

**Keywords:** Acute amiodarone pulmonary toxicity, clinical vignette, lung resection

## Abstract

Amiodarone is one of the most frequently prescribed antiarrhythmic agents. Despite its widespread use, it is associated with systemic side effects. Pulmonary toxicity, the most severe adverse effect of amiodarone, has usually been described in the context of chronic amiodarone use. We report a case of an 80-year-old male presenting acutely following right upper lung lobe resection for stage 1b adenocarcinoma. He developed atrial fibrillation on postoperative day four and received 12.5 g of amiodarone within a 12 day period. On presentation, he had new bilateral lung opacities and a 35% absolute decline in the predicted diffusion capacity for carbon monoxide. Pulmonary embolism was ruled out on chest computed tomography. Amiodarone was discontinued and prednisone was initiated. Despite initial improvement, he suffered from multiple hypoxemic episodes until his death in the fourth month. In a subset of patients undergoing thoracic surgery who are intubated and require high levels of oxygen, the risk of amiodarone lung toxicity increases and patients may present acutely.

## INTRODUCTION

Amiodarone is an iodinated benzofuran derivative that is used for the effective management of a wide variety of arrhythmias. It is associated with side effects including pulmonary toxicity, hepatotoxicity, cardiotoxicity, thyroid dysfunction and corneal microdeposits (1-3). Of these, pulmonary toxicity occurring in 2 to 10% of treated patients is the most severe adverse effect accounting for the greatest proportion of amiodarone associated deaths (3-7).

Amiodarone pulmonary toxicity (APT) usually occurs in the setting of high cumulative doses over a period of months to years (8). However, there have been reports of APT with lower doses of amiodarone presenting acutely within days to weeks of treatment initiation (9, 10). Other risk factors for APT include increasing age, preexisting lung disease, thoracic surgery and pulmonary angiography (2, 10).

The mechanism of amiodarone induced lung damage is not completely understood (11). The prevailing theory suggests a direct cytotoxic lung injury and an indirect hypersensitivity reaction (8). Due to its lipophilic nature, amiodarone accumulates in the phospholipid bilayer and thus impairs cellular function. In addition, amiodarone promotes the production of toxic oxygen radicals which can also cause direct cellular injury (12). This proposed pathogenesis may have implications for clinical practice. In a subset of patients undergoing thoracic surgery who are intubated and require high levels of oxygen, it has been postulated that the risk of developing APT is further heightened due to amiodarone sensitization to high concentrations of oxygen (13, 14).

We report a case of acute APT following lung resection presenting within a few days of therapy initiation and fatality occurring within four months. Our objectives were: (1) Recognize that acute APT can occur any time after commencement of amiodarone and it is a diagnosis of exclusion; (2) Recognize the increased risk of acute APT following thoracic surgery.

## CASE REPORT

An 80-year-old male with a 15-pack-year cigarette smoking history and coronary artery disease underwent a right upper lobectomy for lung adenocarcinoma (T2A N0 M0, Stage 1b). The postoperative (postop) course was complicated by subcutaneous emphysema which necessitated prolonged intubation. On postop day four, he developed paroxysmal atrial fibrillation with rapid ventricular rate and was loaded with IV amiodarone for rhythm control at a rate of 0.5 mg/min. On postop day eight, it was then converted to 1.2 g in three divided doses orally. He was transferred to a rehabilitation center on postop day 12 in a relatively stable condition.

**Figure 1:**
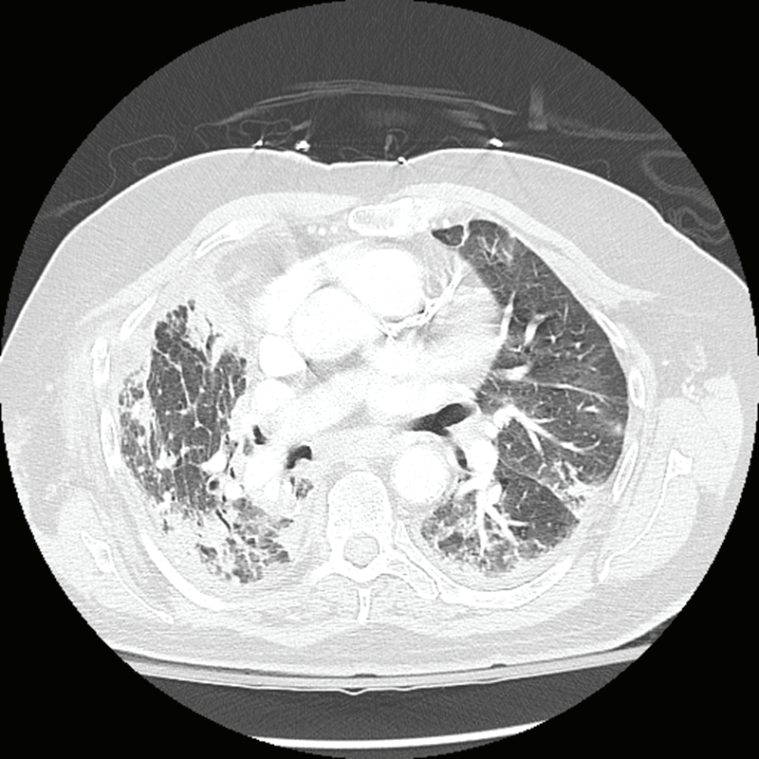
Computed tomography of the chest showing bilateral airspace opacities.

On postop day 15, patient presented to the hospital with severe dyspnea, dry cough and syncope. He denied chest pain, diaphoresis, nausea or fever. He had received a total dose of 12.5 g amiodarone in the preceding 12 days. There were no known environmental exposures. On physical examination, patient had diffuse dry crackles in both lung fields and no wheezes. Neck veins were not distended and there was no pedal edema. Arterial blood gas showed hypoxemia without CO_2_ retention. Cardiac enzymes were normal and there were no acute EKG changes. Laboratory studies showed a leukocytosis and elevated ESR. Chest CT scan revealed new bilateral airspace opacities predominantly affecting the lung bases (Figure 1). Pulmonary embolism was excluded. Angiotensin converting enzyme level was normal and collagen vascular work up, including ANA, RF, ANCA and Scl-70 was negative. Blood culture did not grow any organisms. Postop pulmonary function test revealed severe restrictive disease evidenced by a marked decline in the diffusion capacity for carbon monoxide (DLCO) as shown in Table 1. Transthoracic echocardiogram showed normal valvular and left ventricular function with elevated pulmonary artery (PA) pressure (80 mmHg). Cardiac catheterization completed seven months ago showed normal PA pressure. There was no low suspicion for sleep apnea and hence a sleep study was not done.

**Table 1:** Preoperative and postoperative lung function tests

	Preoperative	% Predicted	Postoperative^a^	% Predicted

**FEV1^b^ (L)**	2.19	71	1.24	41
**FVC^c^ (L)**	2.86	71	1.61	39
**FEV1/FVC (%)**	77	N/A^f^	81	N/A^f^
**TLC^d^ (L)**	4.45	66	3.13	42
**DLCO^e^ (ml/mmHg/min)**	13.50	59	6.50	24

a 20 days post right upper lobe resection and 16 days post amiodarone initiation;

b Forced expiratory volume in 1s;

c Forced vital capacity;

d Total lung capacity;

e Diffusion capacity for carbon monoxide;

f Not applicable.

Amiodarone was discontinued and he was started on prednisone due to suspicion of APT. He improved symptomatically and radiographically, however he was still requiring 3 L of intranasal oxygen. On postop day 23, he was discharged back to the rehabilitation center on oxygen and was placed on one month prednisone taper. Due to initial improvement in clinical status and absence of indication for continuation of amiodarone (no atrial fibrillation on telemetry during hospital stay), it was decided not to obtain a lung biopsy as the risk outweighed the benefit.

On postop day 52 (four days after completing steroid therapy), he again presented to the hospital with similar complaints of severe dyspnea with high oxygen requirements. Radiological findings were improved but not back to baseline. Pulmonary embolism was again excluded. He underwent bronchoscopy which revealed normal bronchial mucosa. Microbiologic analysis of bronchial washing showed a neutrophilic leukocytosis, was negative for acid fast bacilli and there was no growth on culture. Bronchial washing cytology was negative for malignancy. Clinically, he improved and he was discharged on a prolonged course of prednisone. He subsequently had multiple admissions for hypoxemia until he died on postop day 112.

## DISCUSSION

APT is the most severe adverse effect associated with the use of amiodarone (4, 12). Pulmonary fibrosis following APT is irreversible, therefore anticipation and early detection of this potentially fatal side effect is important (13, 15). Onset of APT has usually been observed after several months or years of amiodarone use. In the above patient and a handful of other cases reported in literature, pulmonary toxicity can occur within a few days to weeks particularly in patients undergoing surgery (7, 9, 10, 14, 16). Handschin *et al* in 2003 reported a case of amiodarone-induced pulmonary toxicity following lung resection presenting with severe dyspnea within three days of amiodarone initiation and subsequently developing adult respiratory distress syndrome (ARDS) on the 20^th^ day after a total amiodarone dose of 9 g (10). Similarly, Van Mighem *et al* in 1994 reported three cases of lung toxicity following lung resection presenting with ARDS within 72 hours of amiodarone use (amiodarone doses between 2 and 4 g) (13). Our case did not develop ARDS, however he presented in the same timeframe within 12 days after receiving a total amiodarone dose of 12.5 g.

The clinical presentation of APT varies from pneumonia-like, ARDS, diffuse alveolar hemorrhage to solitary pulmonary mass (12, 15). It is unclear what determines the particular mode of presentation in individual patients but ARDS occurs almost exclusively following thoracic surgery (14, 17, 18). The clinical findings are non-specific and it is a diagnosis of exclusion. The diagnosis of APT needs to be considered in high risk patients on amiodarone irrespective of dose, presenting with new or worsening respiratory symptoms or signs, new chest radiographic abnormalities, decline in DLCO > 20%, marked CD8+ lymphocytosis in bronchoalveolar lavage fluid, typical pathology on lung biopsy, or improvement in lung manifestations following amiodarone withdrawal. The presence of 3 or more of the above factors is suggestive of a diagnosis of APT (8, 15). KL-6, a mucin-like glycoprotein produced by type II pneumocytes has potential for being a marker of APT (19).

Prevention of toxicity starts with using the lowest possible amiodarone dose for rhythm control. A recent 2013 review by Range et al of randomized clinical trials for the management of supraventricular tachycardia showed appropriate loading doses of 600 to 800 mg/day for two to three weeks before reducing to 200 mg/day maintenance doses (20). Management of APT includes discontinuation of amiodarone and may require steroid treatment for greater than six months. As in the case presented, tapering steroids too early may lead to symptom recurrence.

Fatality from APT is low as response to steroids occurs in up to 75% of treated patients (21). Fatality is more likely in ARDS cases with mortality rates as high as 50% (15). A recently published observational study found that the severity of APT is related to the rapidity of onset of lung damage (22). Review of literature specifically for APT cases following lung surgery is consistent with the above findings and shows that fatality occurs within days to weeks of the initial presentation of ARDS (13, 16). In our case, despite the acute presentation, the patient did not succumb to the disease until later in the fourth month. This may be due to the fact that he did not rapidly develop extensive lung damage and as such did not have ARDS; hence, time to fatality was longer than previously reported in the literature.

Risk factors in our case include old age, preexisting lung disease (23), thoracic surgery, intubation and amiodarone dose greater than 400 mg/day (4). In a subset of patients undergoing thoracic surgery who are intubated and require high levels of oxygen, it has been postulated that the risk of developing APT is further heightened due to increased susceptibility for lung damage (13, 14). The reasons for this are manifold. In addition to the lung insult from surgery, these patients have underlying lung diseases and are likely to require higher levels of oxygen postop. One of the mechanisms of amiodarone-induced lung toxicity is the production of toxic oxygen radicals which can cause direct cellular injury (12). Hence, the availability of high amounts of oxygen, pre-existing lung disease and lung insult from surgery provides a suitable environment for amiodarone toxicity. There are a number of case studies that have reported an acute ARDS-like presentation in postop patients on amiodarone (10, 16).

Due to its efficacy, amiodarone will continue to be one of the commonly prescribed antiarrhythmic agents until safer and effective agents are identified. Physicians need to be aware of this potentially acute and fatal complication of amiodarone use. In patients with multiple risk factors including thoracic surgery, there needs to be a consideration of the risks and benefits of amiodarone use prior to treatment initiation.
